# Exercise‐conditioned plasma attenuates nuclear concentrations of DNA methyltransferase 3B in human peripheral blood mononuclear cells

**DOI:** 10.14814/phy2.12621

**Published:** 2015-12-10

**Authors:** Steven Horsburgh, Stephen Todryk, Christopher Toms, Colin N. Moran, Les Ansley

**Affiliations:** ^1^ Faculty of Health and Life SciencesNorthumbria UniversityNewcastle Upon TyneEngland; ^2^ Research DepartmentBritish College of Osteopathic MedicineLondonEngland; ^3^ Health and Exercise Sciences Research GroupUniversity of StirlingStirlingScotland

**Keywords:** DNA methylation, epigenetics, inflammation

## Abstract

DNA methylation is modifiable by acute and chronic exercise. DNA methyltransferases (DNMT) catalyze this process; however, there is a lack of literature concerning the specific mechanisms by which exercise‐induced modifications occur. Interleukin 6 (IL‐6) stimulation of various cell lines has been shown to augment DNMT expression and nuclear translocation, which suggests a possible pathway by which exercise is able to elicit changes in epigenetic enzymes. The present study sought to elucidate the response of the *de novo* methyltransferases DNMT3A and DNMT3B to circulatory factors found in plasma isolated from whole blood before and after 120‐min of treadmill running at an intensity of 60% of individual velocity at $\dot{V}O_{2\max}$ (v$\dot{V}O_{2\max}$) interspersed with 30‐sec sprints at 90% of v$\dot{V}O_{2\max}$ every 10‐min. Peripheral blood mononuclear cells (PBMCs) isolated from a resting participant were incubated with plasma isolated from exercising participants (*n* = 10) or recombinant IL‐6 (rIL‐6), followed by nuclear protein extraction and quantification of DNMT3A and DNMT3B concentrations. Nuclear concentrations of DNMT3B significantly decreased following the experimental protocol (*P *=* *0.03), with no change observed in DNMT3A (*P *=* *0.514).Various concentrations of rIL‐6 caused an elevation in both DNMT3A and DNMT3B nuclear concentration compared with the blank control. The conflicting results between exercising and rIL‐6 conditions suggests that IL‐6 does regulate DNMT nuclear transport, however, other plasma mediators may also exert significant influence on the nuclear concentrations of these enzymes.

## Introduction

Epigenetic modifications, such as DNA methylation and histone tail methylation, acetylation, and phosphorylation, are processes by which functional changes to DNA occur independently from alterations to the nucleotide sequence. DNA methylation is the most abundantly studied epigenetic modification, and is characterized by the DNA methyltransferase (DNMT)‐regulated addition of a methyl group to the nucleotide cytosine creating 5‐methylcytosine. This process occurs predominantly at Cytosine‐phosphate‐Guanine (CpG) dinucleotides; cytosine and guanine separated by a single phosphate bond which are statistically underrepresented in the genome (Lander et al. 2001). CpG islands, present in up to 72% of gene promoter regions (Saxonov et al. 2006), are short sequences, typically around 1000 base pairs long, and differ from usual genomic CpGs due to their low level or complete absence of methylation (Deaton and Bird 2011). Hypomethylation at these CpG‐rich gene promoter regions is often associated with active gene transcription. Conversely, promoter region hypermethylation is usually associated with transcriptional silencing, of which the inhibition of transcription factor binding and the recruitment of methyl‐CpG binding proteins, which repress the chromatin structure, are key mechanisms (Auclair and Weber 2012). A number of DNMTs regulate the methylation process; DNMT1 methylates hemimethylated DNA, and therefore, has an important role with regard to the maintenance of methylation through cell division. DNMT3A and DNMT3B, on the other hand, show preference toward unmethylated CpG dinucleotides and are both involved in *de novo* methylation. All three of these DNMTs are essential in mammalian development, as demonstrated by the death of DNMT‐deficient mice shortly after birth (Li 2002).

Exercise is known to promote a multitude of health benefits, including decreased risk of cancer (Lee 2003), cardiovascular disease (Thompson et al. 2003), and type 2 diabetes mellitus (Colberg et al. 2010); however, the involvement of epigenetics in mediating these benefits remains to be fully understood. The study of habitual physical activity and long‐term exercise interventions has received some research attention in the context of epigenetic modifications, with studies showing that chronic exercise can alter the DNA methylome of various tissues, including skeletal muscle, adipose tissue, leukocytes, and sperm (Nitert et al. 2012; Rӧnn et al. 2013; Denham et al. 2015a,b). Unfortunately, however, there is a relative lack of literature concerning the acute effect of exercise on DNA methylation. Barrès et al. (2012) reported that a single $\dot{V}$O_2peak_ test conducted on a cycle ergometer was able to cause global hypomethylation of *vastus lateralis* skeletal muscle in a cohort of sedentary young men and women. They also assessed methylation of a number of genes which are normally upregulated following an acute exercise bout, using methylated DNA immunoprecipitation and quantitative PCR. Hypomethylation occurred at the peroxisome proliferator‐activated receptor gamma coactivator 1 alpha (*PGC‐1α*), pyruvate dehydrogenase lipoamide kinase isozyme 4 (*PDK4*), peroxisome proliferator‐activated receptor delta (*PPARδ*), transcription factor A mitochondrial (*TFAM*), and citrate synthase (*CS*) gene promoter regions, whereas the methylation status of muscle‐specific genes, myocyte‐specific enhancer factor 2A (*MEF2A*) and myogenic differentiation 1 (*MYOD1*), remained unaltered. This suggests that the exercise‐induced upregulation (mRNA and protein level) of genes involved in mitochondrial biogenesis and substrate metabolism (Coffey and Hawley 2007) is due to epigenetic modification. Given the aforementioned benefits of regular physical activity and decreased disease risk, exercise‐induced gene promoter hypomethylation may be a mechanism by which exercise exerts salubrious effects.

Furthermore, a recent study conducted by Robson‐Ansley et al. (2014) found that plasma interleukin‐6 (IL‐6) may be linked with DNA methylation. The researchers investigated the effect of a 120‐min treadmill run at 60% of v$\dot{V}O_{2\max}$ interspersed with sprints at 90% of v$\dot{V}O_{2\max}$ for the last 30‐sec of every 10‐min (previously shown to induce elevations in plasma IL‐6; Walshe et al. 2010), followed by a 5‐km time trial, on subsequent changes in DNA methylation of peripheral blood mononuclear cells (PBMCs) as measured by the Infinium Human Methylation 27k beadchip (Illumina, San Diego, CA, USA). Despite no significant change in global DNA methylation, the exercise‐induced increase in plasma IL‐6 concentration was significantly correlated with the methylation status of 11 genes (signaling lymphocytic activation molecule 1 [*SLAMF1*], interleukin‐1 receptor‐associated kinase 3 [*IRAK3*], LIM domain binding 2 [*LDB2*], transmembrane protein 156 [*TMEM156*], FC receptor‐like 2 [*FCRL2*], cyclin‐dependent kinase 9 [*CDK9*], signaling threshold‐regulating transmembrane adaptor 1 [*SIT1*], EFG domain‐specific O‐linked N‐acetylglucosamine transferase [*AER61*], recombination‐activating gene 2 [*RAG2*], phosphoseryl‐TRNA kinase [*C10orf89*], CD40 ligand [*CD40LG*]), a number of which are regulators of activities involving B and T cells, while *IRAK3* is a key inhibitor of inflammation associated with the metabolic syndrome and obesity. IL‐6 likely modifies DNA methylation via manipulation of DNMT expression and cellular translocation; *in vitro* IL‐6 stimulation of K562 (human erythromyeloblastoid leukemia) and A549 (human adenocarcinomic alveolar basal epithelial) cells increased DNMT1 expression via Janus kinase/Signal transducer and activator of transcription (JAK2/STAT3) activation (Hodge et al. 2001; Liu et al. 2015), while similarly, Hodge et al. (2007) reported that augmented DNMT1 activity may be caused by protein kinase B (AKT)‐dependent phosphorylation of the DNMT1 nuclear localization signal, thus allowing nuclear translocation, following stimulation of HCT116 (human colorectal carcinoma) cells with IL‐6. As to whether IL‐6 directly interacts with DNMTs in PBMCs has yet to be reported however.

Many studies (Zhang et al. 2012; Shaw et al. 2014; Denham et al. 2015b) have used blood leukocytes in order to investigate CpG methylation in response to habitual physical activity or exercise, as they may be useful biomarkers of systemic changes (Denham et al. 2015b). However, considerable variation in methylation exists between the various cell populations (Reinius et al. 2012), therefore, PBMCs not only represent a more homogenous cell population than whole blood leukocytes, but should also reflect systemic changes. Also, given that aberrant DNA methylation in PBMCs is linked to various cancers (Kitkumthorn et al. 2012; Friso et al. 2013; Ganapathi et al. 2014), schizophrenia (Auta et al. 2013), type 2 diabetes mellitus (Simar et al. 2014), Alzheimer's disease (Di Francesco et al. 2015), vitiligo (Zhao et al. 2010), idiopathic thrombocytopenic purpura (Tao et al. 2008), and rheumatoid arthritis (Nile et al. 2008), they appear to be a relatively noninvasive and inexpensive tissue, in which exercise‐induced epigenetic modifications may have beneficial effects on disease pathogenesis.

The aim of the present study, therefore, was to characterize potential changes in PBMC nuclear concentrations of the *de novo* DNA methyltransferases DNMT3A and DNMT3B caused by circulatory factors located in the plasma following a protocol shown to elicit an acute, transient increase in plasma IL‐6. It was hypothesized that nuclear concentrations of DNMT3B would be significantly decreased following the exercise protocol, consistent with results reported by Laye and Pedersen (2010).

## Methods

### Subjects

Ten recreationally active males were recruited from Northumbria University to take part in the exercise trial. Mean (standard deviation) characteristics are reported in Table 1. Participants gave written informed consent, and all methods were approved by the Northumbria University Ethics Committee. All PBMCs were isolated from a resting, recreationally active participant who was recruited under the same criteria as other participants. Furthermore, his characteristics fit within the standard deviation of the exercising participants' age, height, weight, and BMI.

**Table 1:** Subject characteristics (*n* = 10)

Characteristic	Mean (SD)
Age (years)	24 (3)
Height (cm)	178.5 (5.6)
Weight (kg)	77.5 (8.8)
Body mass index (kg·m^−2^)	24.3 (2.6)
$\dot{V}O_{2\max}$ (mL·kg^−1^·min^−1^)	51.9 (6.7)

### Experimental protocol

The following protocol has previously been reported elsewhere (Walshe et al. 2010) and has been shown to elicit a significant transient increase in plasma IL‐6.

#### Familiarization

Participants first attended the laboratory in order to be familiarized with the study protocol. This involved completing the full experimental protocol, as described below, without blood samples being taken.

#### $\dot{V}{\text{O}}_{2\max}$ assessment

Seven days after the initial familiarization, maximal oxygen uptake ($\dot{V}O_{2\max}$) was quantified using a motorized treadmill (Pulsar, h/p/cosmos, Germany). Treadmill speed was set to 12 km·h^−1^ on a 1% gradient and increased by 1 km·h^−1^ every 3‐min until the participant reached volitional exhaustion. An online breath‐by‐breath analyzer (Metalyzer 3B, Cortex, Germany) measured expired gas, which was used to calculate running velocity at v$\dot{V}O_{2\max}$. Heart rate was recorded via short‐range telemetry (Polar RS400, Finland) during the last 30‐sec of each stage. The test was considered maximal if two of the following criteria were met: a heart rate greater than 90% of the age predicted maximum (220 beats·min^−1^ − age), a change in $\dot{V}O_{2\max}$ of less than 2 mL kg^−1^·min^−1^ in the final two stages, or a respiratory exchange ratio of 1.15 or greater.

#### Main trial

The main experimental trial was conducted following a rest period of 7 days. Participants were instructed to abstain from exercise, alcohol, and caffeine for the 24‐h period prior to testing. Participants arrived at 0900 following an overnight 12‐h fast, during which they were allowed to consume water *ad libitum*. Height and weight were measured using standard procedures, and 22.5 mL of blood was then collected into EDTA containing vacutainers (Becton Dickinson, Oxford, UK) from a vein in the antecubital fossa of the forearm. The main protocol required participants to run at 60% of v$\dot{V}O_{2\max}$ for 120‐min, interspersed with sprints at 90% of v$\dot{V}O_{2\max}$ for the last 30‐sec of every 10‐min. Immediately upon completion of the run, a further blood sample was taken using the same procedure as previously described. Pre‐ and postrun blood samples were centrifuged at 1700 *g* for 10‐min at 4°C in order for plasma to be separated. Samples were immediately stored at −80°C for later analysis.

### Interleukin‐6 assay

A QuantiGlo Human IL‐6 ELISA (R&D Systems, Minneapolis) was used in order to quantify pre‐ and post‐exercise plasma concentrations of IL‐6 as per the standard manufacturer's guidelines. Samples were assayed in duplicate, including positive controls and various dilutions of the IL‐6 standard which were used to plot a standard curve. Assay sensitivity was 0.35 pg·mL^−1^, and the assay detection range was between 0.480 and 1500 pg·mL^−1^. Intra‐assay coefficient of variation was calculated as 4.3%.

### Peripheral blood mononuclear cell viability and nuclear protein extraction

In order to elucidate the role of circulatory factors found in plasma, exercise‐induced changes to the fractional cellular composition of PBMC samples (Connolly et al. 2004) were controlled for by utilizing an identical cell sample isolated from a single, resting participant in all experimental conditions (described earlier). Furthermore, the existence of single nucleotide polymorphisms of genes involved in the DNA methylation cycle has led to the assertion that there may be a genetic susceptibility to epigenetic modification (Terruzzi et al. 2011), thus, a single set of cells is necessary to be able to isolate the specific effects of exercise‐conditioned plasma. Preliminarily, PBMC viability following incubation with exercise‐conditioned plasma was calculated. Whole blood was collected into six 10 mL lithium heparin vacutainer tubes (Becton Dickinson, Oxford, UK). PBMCs were isolated using centrifugation of a LeucoSep centrifuge tube (Greiner Bio‐One, Frickenhausen, Germany) containing Lymphoprep (Stemcell Technologies, Vancouver, Canada). Following counting of the cells, they were diluted accordingly in order to yield 4 × 10^6^ cells·mL^−1^. About 250 *μ*L of PBMCs (1 × 10^6^ cells) were treated with 100 *μ*L of either pre‐exercise plasma, post‐exercise plasma, or fetal calf serum (FCS), in addition to 150 *μ*L of RPMI‐1640 medium and penicillin/streptomycin/glutamine solution (Sigma Aldrich, Missouri), followed by incubation at 37°C for 4‐h. Each condition was performed in triplicate. Before and after incubation, the cells were mixed with trypan blue solution (Sigma Aldrich, Missouri) in a 1:1 ratio, followed by 10 *μ*L of the resultant solution being pipetted into the upper chamber of an Improved Neubauer cell counting chamber (Hawksley, Sussex, United Kingdom). The number of live and dead cells was then counted using a Nikon TMS‐F inverted microscope (Nikon, Tokyo, Japan).

In order to extract nuclear proteins, 250 *μ*L of PBMCs (1 × 10^6^ cells) were treated with 100 *μ*L of plasma separated from whole blood of the exercising participants and 150 *μ*L of RPMI‐1640 medium and penicillin/streptomycin/glutamine solution (Sigma Aldrich, Missouri), followed by incubation at 37°C for 4‐h. Separately, the same volume and concentration of PBMCs were treated with 100 *μ*L of FCS and various concentrations of recombinant interleukin 6 (rIL‐6): 0, 0.01, 0.1, 1, 10, and 100 ng·mL^−1^. 100 ng·mL^−1^ has previously been shown (Hodge et al. 2005; Foran et al. 2010) to increase DNMT1 expression in a multiple myeloma cell line, while 10 ng·mL^−1^ was utilized by Liu et al. (2015), who reported an increase in DNMT1 expression in a lung cancer cell line. The lowest concentration would be considered to be within the expected physiological range following prolonged endurance exercise. Nuclear protein extraction was then performed as per the manufacturer's guidelines (Episeeker Nuclear Extraction Kit, Abcam, Cambridge, UK).

### Bradford protein assay

Bradford assay was used to quantify the amount of protein per *μ*L of nuclear extract (Quick Start Bradford Protein Assay, Bio Rad, California). The microplate was read at an absorbance of 595 nm (Synergy HT, Bio Tek, Vermont). The average blank value was subtracted from the sample absorbance, and a standard curve was generated in order to quantify protein amount for each nuclear extract.

### DNMT quantification

Quantification of nuclear DNMT3A and DNMT3B enzyme concentrations were performed as per the manufacturer's guidelines (Epiquik DNMT3A/B Assay Kit, Epigentek, New York). All samples were assayed in duplicate, and various dilutions of the respective DNMT standards were used to plot a standard curve. Microplates were scanned at 450 nm with a reference wavelength of 655 nm. Intra‐assay coefficient of variations for DNMT3A and DNMT3B were calculated as 3.95% and 9.92%, respectively.

The following equation was used to quantify enzyme concentration:

$$\text{Concentration}\left(\text{ng}\cdot \text{mg of}\;{\text{protein}}^{-1}\right)=\left(\text{sample OD}-\text{blank OD}\right)/\left(\text{slope}\times \text{protein amount}\right)\times 1000$$

where OD is optical density/absorbance. Slope is generated from the standard curve. Protein amount (*μ*g) as quantified by Bradford assay.

### Statistical analysis

Data were analyzed using IBM SPSS Statistics version 21. Prior to performing parametric testing of the data, the Shapiro–Wilk test of normality was utilized. Non‐normally distributed data were subsequently log^10^ transformed. Paired samples *t*‐tests were used to test for significance of repeated measures data. For independent group analysis, Levene's test was utilized in order to ensure equality of variances, followed by independent samples *t*‐test. Mauchly's test of sphericity and one‐way repeated measures ANOVA were used to analyze differences in nuclear DNMT concentrations following stimulation with rIL‐6. Statistical significance was set as *P *≤* *0.05.

Sample size was calculated using a spreadsheet published by Boston University/Boston Medical Center. At the time of planning of this study there had not been any data published that reported changes in nuclear concentrations of DNA methyltransferase enzymes, and therefore, power calculations were based on initial findings from our laboratory that showed that recombinant human IL‐6 was able to significantly augment nuclear concentrations of these enzymes.

## Results

Mean characteristics of the participants are provided in Table 1. Mean body mass index shows that participants were within the normal healthy range of less than 25 kg·m^−2^. Participants were defined as recreationally active and showed a relatively high level of aerobic fitness, reflected by a mean maximal oxygen consumption of approximately 51.9 mL·kg^−1^·min^−1^.

### Peripheral blood mononuclear cell viability

Table 2 shows that in each condition, there was no statistically significant difference in cell viability before and after incubation for 4‐h at 37°C.

**Table 2:** Percentage of viable peripheral blood mononuclear cells before and after incubation period

Condition	Pre‐incubation (%) Mean (SD)	Post‐incubation (%) Mean (SD)
Pre‐exercise plasma	99.2 (0.7)	98.1 (0.1)
Post‐exercise plasma	98.7 (0.1)	96.9 (2.7)
Fetal calf serum	95.6 (1.5)	97.7 (2.0)

### Exercise‐conditioned plasma cell stimulation

**Figure 1:**
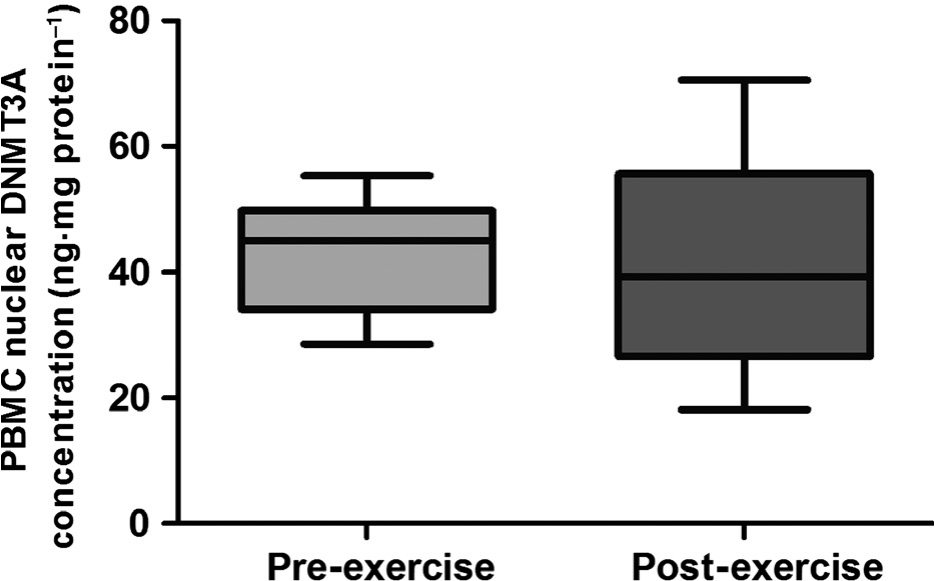
Mean (±95% CI; ±min./max.) peripheral blood mononuclear cell (PBMC) nuclear concentration of DNA methyltransferase 3A (DNMT3A) following stimulation with pre‐ and post‐exercise plasma. A paired sample *t*‐test was used to test significance.

**Figure 2:**
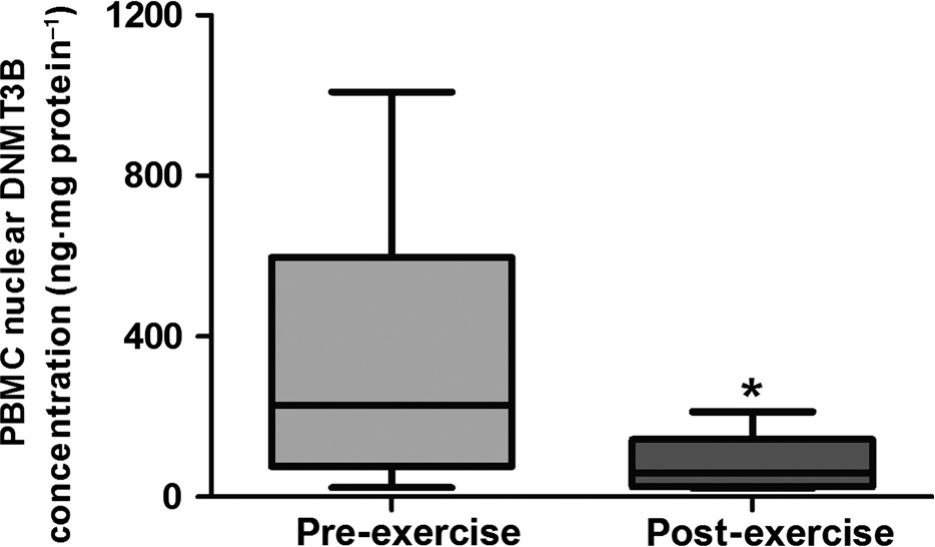
Mean (±95% CI; ±min./max.) peripheral blood mononuclear cell (PBMC) nuclear concentration of DNA methyltransferase 3B (DNMT3B) following stimulation with pre‐ and post‐exercise plasma. A paired sample *t*‐test was used to test significance (*P <* *0.05).

The effects of cell stimulation with plasma isolated following 120‐min of treadmill running, on subsequent changes in nuclear concentrations of the *de novo* DNA methyltransferases DNMT3A and DNMT3B are illustrated visually in Figures 1 and 2. No change was observed in nuclear concentration of DNMT3A following the exercise stimulus, with mean (SD) “pre” and “post” concentrations measured at 43.1 (8.7) and 41.5 (17.4) ng·mg protein^−1^, respectively (*P *=* *0.736).

Conversely however, mean (SD) nuclear concentrations of DNMT3B significantly decreased from 365.9 (363.6) to 87.2 (73.3) ng·mg protein^−1^ (*P *=* *0.042) – an approximate 76% reduction immediately following the exercise bout.

Baseline concentrations of DNMT3B were significantly higher than DNMT3A (*P *=* *0.02), showing that DNMT3B is more immediately abundant within PBMC nuclei.

### Circulatory IL‐6 concentration

**Figure 3:**
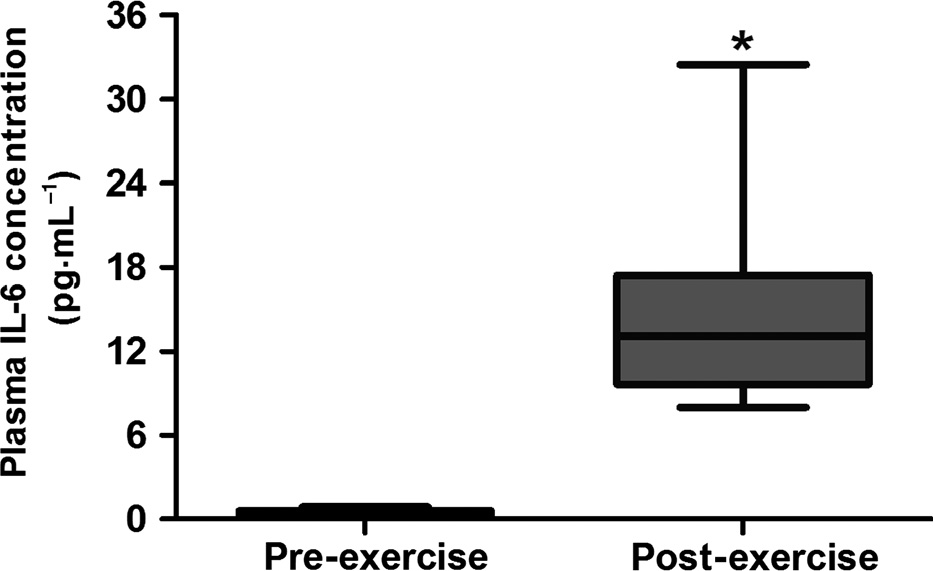
Mean (±95% CI; ±min./max.) pre‐ and post‐exercise plasma concentrations of interleukin 6 (IL‐6). A paired sample *t*‐test was used to test significance (*P <* *0.05).

Plasma concentrations of IL‐6 were also quantified. Figure 3 shows that mean (SD) systemic concentrations significantly increased from 0.4 (0.3) to 14.9 (7.4) pg·mL^−1^ – a 35‐fold increase “pre” to “post” exercise (*P *=* *0.005).

### Recombinant IL‐6 cell stimulation

In order to isolate the possible effects of IL‐6 on subsequent changes in DNMT3A and DNMT3B nuclear concentrations, cells were also stimulated with various concentrations of rIL‐6 separately from exercise‐conditioned plasma. Results are represented in Table 3.

**Table 3:** Mean (SD) peripheral blood mononuclear cell (PBMC) nuclear concentration of DNA methyltransferases 3A and 3B (DNMT3A/DNMT3B) following stimulation with various concentrations of recombinant interleukin 6 (rIL‐6) (0, 0.01, 0.1, 1, 10, and 100 ng·mL^−1^)

rIL‐6 concentration (ng·mL^−1^)	Mean (SD) Nuclear DNMT3A Concentration (ng·mg protein^−1^)	Mean (SD) Nuclear DNMT3B Concentration (ng·mg protein^−1^)
0	3.3 (0.9)	32.8 (0.8)
0.01	28.9 (3.2)^a^	46.1 (16.7)
0.1	44.2 (1.1)^a^, ^b^	88.8 (3.2)^a^
1	53.0 (4.5)^a^	90.9 (11.3)
10	60.3 (1.6)^a^	205.1 (7.7)^a^, ^b^, ^c^, ^d^
100	80.2 (5.7)^a^	458.7 (28.7)^a^, ^b^, ^c^, ^d^, ^e^

A repeated measures ANOVA was used to test statistical significance.

a Significantly greater than 0 (*P *<* *0.05).

b Significantly greater than 0.01 (*P *<* *0.05).

c Significantly greater than 0.1 (*P *<* *0.05).

d Significantly greater than 1 (*P *<* *0.05).

e Significantly greater than 10 (*P *<* *0.05).

All concentrations of rIL‐6 caused a significant increase in nuclear DNMT3A concentration when compared with the blank control (0.01, 0.1, 1, 10, and 100 ng·mL^−1^ concentrations had *P* values of 0.041, 0.002, 0.033, 0.02, and 0.038, respectively), while 0.1 ng·mL^−1^ resulted in a significantly greater DNMT3A concentration than 0.01 ng·mL^−1^ (*P *=* *0.023). Despite the results visually appearing to increase in a dose–response manner, no other interactions passed the statistically significant threshold of 0.05.

Nuclear DNMT3B concentrations were significantly higher in 0.1, 10, and 100 ng·mL^−1^ conditions, compared with the blank control (*P *=* *0.032, 0.022, and 0.031, respectively). Furthermore, the 10 ng·mL^−1^ condition caused a significant augmentation when compared with 0.01, 0.1, and 1 ng·mL^−1^ (*P *=* *0.025, 0.018, 0.014, respectively). Finally, mean DNMT3B concentration following 100 ng·mL^−1^ of rIL‐6 was significantly greater than all other rIL‐6 conditions (*P *=* *0.013, 0.031, 0.021, and 0.037, respectively).

## Discussion

This is the first study to investigate changes in the concentrations of the *de novo* DNA methyltransferases DNMT3A and DNMT3B following stimulation of PBMCs with plasma isolated before and after an acute bout of intense treadmill exercise. While DNMT3A concentrations remained unaltered, a significant reduction in the nuclear concentration of DNMT3B was observed which is consistent with previous findings showing decreased DNMT3B mRNA in *vastus lateralis* skeletal muscle samples following exercise (Laye and Pedersen 2010). These data suggest that the two *de novo* methyltransferases have distinct roles in response to exercise, and highlights a possible mechanism by which exercise may be able to acutely alter levels of DNA methylation. Furthermore, to our knowledge, no study has yet investigated the response of nuclear DNMT concentrations in PBMCs following rIL‐6 stimulation.

The significant increase in plasma IL‐6 immediately following the exercise bout suggests that nuclear transport or transcription of DNMT3A is unlikely to be solely regulated by IL‐6 mediated signaling pathways immediately upon cessation of exercise, in contrast to the response of DNMT3B. The posit that DNMT3A and DNMT3B possess diverse functions following an acute exercise stimulus may be supported by findings which demonstrate that the enzymes are differentially expressed during development; DNMT3B is the primary active enzyme during earlier embryonic stages such as implantation, whereas DNMT3A expression is greater in the later stages of embryonic development (Okano et al. 1999) and during methylation of maturing gametes (Hara et al. 2014).

The observed decrease in nuclear concentration of DNMT3B could be due to two mechanisms: downregulation of gene transcription and/or enhanced nuclear export. Laye and Pedersen (2010) reported a 50% decrease in skeletal muscle DNMT3B mRNA following 3 h of cycling at 60% of $\dot{V}O_{2\max}$. Furthermore, ionomycin (a Ca^2+^ ionophore) stimulation of differentiated myotubes, but not proliferating myoblasts, caused attenuation of both DNMT3A and DNMT3B mRNA by approximately 40%. The 75% decrease in nuclear concentration of DNMT3B within the present study suggests nuclear export of the enzymes may also be occurring concurrently with transcriptional downregulation; 60‐min of cycling has previously been shown to significantly decrease nuclear concentrations of the class IIa histone deacetylases HDAC4 and HDAC5 in skeletal muscle, possibly due to enhanced nuclear export (McGee et al. 2009) via Ca^2+^/calmodium‐dependent protein kinase (CaMK) signaling (McKinsey et al. 2000). Since skeletal muscle CaMKII activity can be augmented by muscular contraction in an intensity‐dependent manner (Rose et al. 2006), when combined with reported contraction‐induced DNA hypomethylation of mouse skeletal muscle (Barrès et al. 2012; Lucas et al. 2012), this suggests a mechanistic link between exercise, specifically muscular contraction, and nuclear export of epigenetic enzymes. Whether epigenetic signaling within peripheral blood cells is the same as within muscle in response to exercise remains to be elucidated.

If the observed decrease in nuclear concentration of DNMT3B did indeed translate into DNA hypomethylation, this would be in contrast to the results reported by Robson‐Ansley et al. (2014), who found no differences in global or gene‐specific DNA methylation following an identical protocol. However, the participants used by Robson‐Ansley et al. (2014) were trained, as opposed to the recreationally active participants and cell donor utilized within the present study. It is possible, therefore, that endurance training‐associated increases in CaMKII (Rose et al. 2007) may attenuate the acute epigenetic response to a further single exercise bout. Furthermore, the Infinium Human Methylation 27k beadchip was utilized in this study which only covers 27, 578 CpG sites across 14, 495 genes, which may explain the lack of detectable changes.

In contrast to the response of DNMT3A and DNMT3B to acute exercise‐conditioned plasma, following rIL‐6 stimulation we observed an elevation of both DNMT3A and DNMT3B nuclear concentrations. IL‐6‐induced AKT phosphorylation of the DNMT1 nuclear localization signal, as previously reported by Hodge et al. (2007), may explain the mechanistic link between IL‐6 stimulation and increased enzyme concentration within the nucleus. Hodge et al. (2005) and Foran et al. (2010) both found that stimulation of multiple myeloma or colorectal carcinoma cell lines with the same concentration of rIL‐6 as utilized within the present study (100 ng·mL^−1^) resulted in significant increases in DNMT1 mRNA. There was no quantification of mRNA levels within our study however, and therefore IL‐6 induced transcriptional upregulation is a speculative mechanism.

The conflicting outcomes between the exercise and rIL‐6 stimulation data suggest that molecules found in the plasma other than IL‐6 could have profound influences on nuclear concentrations of DNMTs. Prostaglandin E_2_ (PGE_2_), a lipid autocoid derived from arachidonic acid, is an important mediator in the acute inflammatory response and can regulate IL‐6 expression in various cell types (Hinson et al. 1996; Williams and Shacter 1997; Inoue et al. 2002; Bagga et al. 2003). Elevated plasma PGE_2_ has previously been reported following a marathon (Demers et al. 1981) and run to exhaustion at 80% of VO_2max_ (Venkatraman et al. 2001), therefore given the intensity of the exercise bout in the present study, it is possible that plasma PGE_2_ was elevated, contributing to the augmentation of circulating IL‐6. Furthermore, expression of Sp1 and Sp3 transcription factors, known to regulate DNMT3A (Jinawath et al. 2005), have been shown to be upregulated following PGE_2_ stimulation, concomitant with an increase in DNMT3A expression in fibroblasts, and a decrease in DNMT3A and DNMT1 expression in RAW macrophages (Huang et al. 2012). This not only demonstrates the role of PGE_2_ in regulation of epigenetic regulatory enzymes, but also the specificity of changes within different cell lines.

Circulating micro RNAs (miRNAs) are another candidate group of plasma molecules that could influence the expression or transport of DNMTs. Changes in plasma concentrations of a multitude of miRNAs have been reported following acute aerobic (Nielsen et al. 2014), resistance (Sawada et al. 2013), and eccentric (Banzet et al. 2013) exercises. The role of IL‐6 in the modification of DNMT1 expression, which has been discussed elsewhere within this article, could be mediated by miRNA‐148a and miRNA‐152 (Braconi et al. 2010). Furthermore, miRNA‐143 has been shown to target DNMT3A (Ng et al. 2009), and miRNA‐148 targets the DNMT3B protein coding region (Duursma et al. 2008), while miRNA‐29 appears to be involved in the regulation of both DNMT3A and DNMT3B (Fabbri et al. 2007; Garzon et al. 2009; Takada et al. 2009).When combining these data, it appears entirely possible that exercise‐induced changes to plasma miRNAs could have a profound influence on nuclear DNMT transport and/or gene expression and warrants further investigation.

### Limitations

A limitation of the present study was the lack of a direct measure of genomic or gene‐specific methylation, and thus, it is not known whether the attenuation of DNMT3B resulted directly in the modification of DNA methylation. Hypo‐ or demethylation is known to occur passively via downregulation of DNMT1, or actively via upregulation of TET activity (Kohli and Zhang 2013); however, the functional effects of a reduction in DNMT3B are unknown.

The nonstatistically significant increases in nuclear DNMT concentrations following stimulation of PBMCs with rIL‐6 are likely due to the low sample size; cells were treated, incubated, and then assayed in duplicate. Visually, there appears to be a dose–response relationship, however, further investigation using larger sample sizes is required in order to elucidate whether these increases in nuclear DNMT concentration are genuine or anomalous.

An investigation into the distinct functions of DNMT3A and DNMT3B is necessary in order to elucidate how the enzymes are differentially regulated, and whether DNMT3A exhibits a delayed response to exercise, similar to the expression of the enzyme later in embryonic development. Therefore, future research should look to sample at multiple time points, and to perform further cell stimulation studies with additional cytokines or compounds, similar to the study conducted by Barrès et al. (2012).

It must also be noted that due to the utilization of a standardized cell sample from a single participant in all experimental conditions, results may lack generalizability. For example, the cell donor in the present study could be a low or nonresponder due to their particular genotype, possibly accounting for the lack of change in DNMT3A concentration following stimulation with exercise‐conditioned plasma.

## Conclusion

This study shows that circulatory factors found in the plasma following a single bout of endurance exercise are sufficient to decrease nuclear concentrations of DNMT3B, which points to IL‐6 as a contributor to exercise‐induced gene promoter hypomethylation, and thus protein upregulation. The precise signaling molecules and pathways involved in these modifications require much elucidation; however, data suggest that IL‐6 may only be one of a number of mediators involved in the manipulation of DNMT nuclear transport and gene expression.

## Conflict of Interest

None declared.
